# Knowledge, Attitudes, Practices, and Burden During the COVID-19 Pandemic in People with Parkinson’s Disease in Germany

**DOI:** 10.3390/jcm9061643

**Published:** 2020-05-29

**Authors:** Hannah M. Zipprich, Ulrike Teschner, Otto W. Witte, Aline Schönenberg, Tino Prell

**Affiliations:** 1 Department of Neurology, Jena University Hospital, 07743 Jena, Germany; hannah.zipprich@med.uni-jena.de (H.M.Z.); ulrike.teschner@med.uni-jena.de (U.T.); otto.witte@med.uni-jena.de (O.W.W.); aschoenenberg@online.de (A.S.); 2 Center for Healthy Ageing, Jena University Hospital, 07743 Jena, Germany

**Keywords:** knowledge, attitudes, practices, COVID-19, pandemic

## Abstract

**Background:** Adherence to measures that have been adopted during the COVID-19 pandemic is crucial to control the spread of the coronavirus. **Methods:** Semi-structured telephone interviews were performed with 99 patients with Parkinson’s disease (PD) and 21 controls to explore knowledge, attitudes, practices, and burden in order to elucidate nonadherence to preventive measures. **Results:** The majority of patients understood the preventive measures and felt sufficiently informed. Analysis of qualitative answers, however, showed that about 30% of patients had an insufficient level of knowledge, which was not associated with educational level, cognitive disorders, or depression. Changes in behaviour were reported by 73 patients (99% performed at least one specific preventive behavior, and 86.9% have reduced social contacts and stayed home). A closer analysis of qualitative answers showed that 27.3% of patients continued to meet relatives face-to-face almost daily. Anxiety and worries about the current situation were reported by 58.6% of patients; 31.3% complained about a decrease in their mobility since the beginning of the restrictions, mainly because of worsening of PD and because regular therapies (e.g., physiotherapy) were canceled. **Conclusions:** About 30% of PD patients are nonadherent to preventive measures. Use of simple dichotomous questions overestimates adherence to preventive measures in patients with PD.

## 1. Introduction

Severe acute respiratory syndrome coronavirus 2 (SARS-CoV-2), a novel virus causing COVID-19 infection, has led to a deadly pandemic. This virus has poorer outcomes and higher mortality rates in older adults and those with comorbidities or chronic diseases such as Parkinson’s disease [1,2,3]. SARS-CoV-2 appeared in early December 2019 in the city of Wuhan, Hubei, China. Since then, local and national governments have taken unprecedented measures in response to the outbreak of SARS-CoV-2-induced coronavirus disease in 2019 (COVID-19), including quarantining infected individuals and their family members, canceling public transportation, exit controls, travel restrictions, contact restrictions, curfews, school closures, and requiring people to wear mouth and nose masks [4,5]. These measures may have several short-term as well as long-term adverse consequences for people with Parkinson´s disease, such as worsening of motor function and stress-related psychiatric symptoms such as anxiety and depressive mood [3]. However, for successful containment of the spread of the virus, it is essential that people with Parkinson´s disease follow the measures. Three factors, among others, are decisive for adherence to these measures: the knowledge of the population, their attitudes, and practical implementation of the recommendations [6]. The lessons learned from the SARS outbreak in 2003 suggest that knowledge and attitudes toward infectious diseases influence the degree of emotional response in the population. Above all, panic can further complicate attempts to prevent the spread of the disease [7]. In order to facilitate the management of the COVID-19 outbreak there is an urgent need to understand public awareness of COVID-19 and the reasons for nonadherence to measures at this critical moment. Perception of risks is important for human decision making. Regarding behavior, emotions such as fear or the feeling of being threatened also play a role. So far, one study has investigated the knowledge, attitudes, and practices (KAP) of Chinese people with respect to COVID-19 [8]. In that study, the majority of the mostly female and well-educated respondents was well informed about COVID-19 and followed the guidelines. However, their average age was 33.0 years, and the results are not transferable to people with Parkinson´s disease. A recent study using telephone interviews of 100 people with Parkinson´s disease suggested that most patients and caregivers were well informed and were coping well with the pandemic [9]. However, multiple-choice or dichotomous questions such as those in the recent study by Prasad et al. cannot adequately reflect KAP [9]. Moreover, one cannot make valid conclusions about the true rates of adherence to preventive measures, and one has to take into account the sociodemographic circumstances of the patients. For this purpose, qualitative methods are necessary. There are no comprehensive data on KAP about COVID-19 in patients with Parkinson´s disease. Describing KAP about COVID-19 in patients with Parkinson´s disease may help to improve adherence to preventive measures.

## 2. Materials and Methods

### 2.1. Participants and Assessments

This cross-sectional survey was conducted from April 2 to 17, 2020. Patients with Parkinson’s disease who were enrolled in the NeuroGerAdh study (DRKS00016774) between August 2019 and February 2020 were interviewed by telephone. The NeuroGerAdh study is a longitudinal observational study of adherence in patients with neurological disorders. This study was approved by the local ethics committee (approval number 5290-10/17) of the Jena University Hospital, and all patients provided written informed consent.

The semi-structured questionnaire consisted of 22 questions to assess the patient’s current situation and adherence to the ongoing regulations, with four of these questions examining the patient’s knowledge of preventive measures (questions 4, 5, 6, and 7), three capturing their attitude toward the virus (questions 8, 9, and 16), and six exploring practices and behavioral changes regarding COVID-19 (questions 10, 11, 12, 13, 14, and 15). In addition, seven questions were included to evaluate the burden and physical and emotional strain felt by the patients due to the ongoing restrictions (questions 16 to 22). Finally, information was obtained on current restrictions, contact with COVID-19 patients, and experience with quarantine. The questionnaire is given in the Supplementary Table S1).

Knowledge of COVID-19 was measured by questions 7 (knowing the correct function of the Robert Koch Institute) and 9 (knowing the correct aims of measures and current restrictions). Patients were grouped according to their knowledge as having good (knows both Robert Koch Institute and aims of measures), moderate (knows one), or poor (knows neither) knowledge. The Robert Koch Institute is an independent German federal authority for infectious diseases. As a public health care institution, it focuses on the health of the entire population and is the government’s central scientific institution in the field of biomedicine in Germany. In terms of COVID-19 the Robert Koch Institute is continuously monitoring the situation, evaluating all available information, estimating the risk for the population in Germany and providing health professionals with recommendations. Its tasks are, among others, the identification, surveillance and prevention of infectious diseases, monitoring and analyzing long-term public health trends in Germany, performing epidemiological and medical analyses, providing a scientific basis for health-related political decision-making, and informing and advising political decision-makers, the scientific sector and the general public (https://www.rki.de).

For each patient, the following parameters were extracted from the medical records: age, gender, marital status, level of education (high: German Abitur or University; low: German Realschule or General Certificate of Secondary Education, German Hauptschule, or no school), and employment status. Information about cognitive state (Montreal Cognitive Assessment (MoCa)) [10], depressive mood (Beck’s Depression Inventory II (BDI)), motor function (Movement Disorder Society–sponsored revision of the Unified Parkinson’s Disease Rating Scale III (MDS-UPDRS III)) [11], presence of non-motor symptoms (Revised Non-motor Symptoms Questionnaire (NMS-Q)) [12,13], and adherence to medication (Stendal Adherence with Medication Score; SAMS) was extracted from the medical records. The SAMS includes 18 questions forming a cumulative scale (0–72) in which 0 indicates complete adherence and 72 complete nonadherence [14,15]. The SAMS is available online (CC BY NC 3.0 license; https://data.mendeley.com/datasets/ny2krr3vgg/1) [16]. Because of the study design, data on these clinical parameters were obtained from two to six months before the interviews were performed.

One hundred patients with Parkinson´s disease were screened for the study. One potential subject could not be reached by telephone (we made three attempts to call the patients). In addition, 25 elderly patients without Parkinson´s disease were randomly selected from the database as controls; 21 of these patients could be interviewed, and 4 were not reached (sociodemographic characteristics are given in Table 1).

### 2.2. Statistical Analysis

Statistical analysis was performed with the statistical software SPSS 25.0 (SPSS Inc., Chicago, IL, USA). Data were analyzed by descriptive statistics: means, standard deviations, medians, interquartile ranges, frequencies, and percentages. Data were checked for normality by the Shapiro–Wilk test. For comparison of groups, the cohort was split into patients <70 and ≥70 years of age. Correlation between different clinical variables was tested with Spearman correlation for non-normally distributed data. Group comparisons were performed by analysis of variance or the Kruskal–Wallis test with Bonferroni correction and the chi-square test. *p*-Values <0.05 were considered to indicate statistical significance.

## 3. Results

### 3.1. Description of the Cohort

The final sample included 35 women (35.4%) and 64 men (64.6%) with Parkinson’s disease, with a median age of 72 years (IQR, 12 years). The median MDS-UPDRS III score was 26 (IQR, 18), and the mean NMS-Q score was 10.5 (SD, 4.2). Most patients were married, receiving a pension, and had completed middle or high school. The majority (55.6%) lived in larger cities (20,000 to 250,000 inhabitants), and 14.1% lived in villages (<2000 inhabitants). None of the patients were infected with coronavirus. Only two patients reported having had contact with a COVID-19-positive person and were in quarantine.

### 3.2. Knowledge

**Figure 1:**
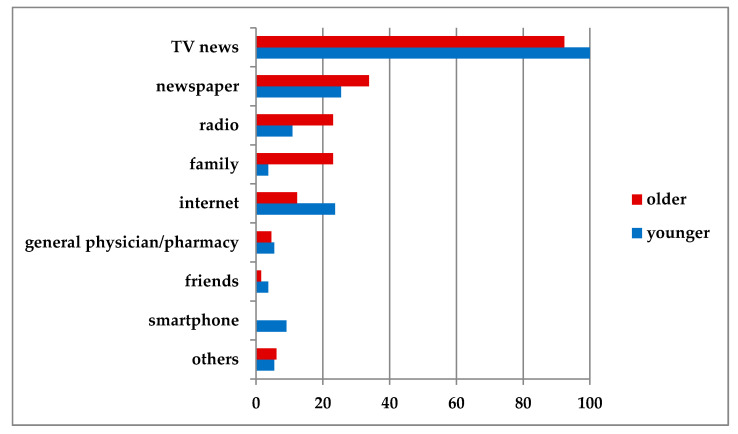
Actively reported sources of information (%). Active reporting means that patients were not given a choice of answers but were free to choose their own.

Most patients reported that they were well or sufficiently informed about COVID-19. Thirty-two (32.3%) reported that they were very well informed, 38 (38.4%) that they were well informed, 16 (16.2%) that they were sufficiently informed, and 5 (5.1%) that they were poorly informed (3 missing). Of note, five patients (5.1%) reported that they received too much information about the virus. Most subjects (patients with Parkinson’s disease and controls) (94.9%) received their information from television news (Figure 1).

Almost half of the patients (47.5%) actively sought additional information on the coronavirus pandemic, mainly via the internet or through acquaintances in the medical profession. Among patients not seeking additional information, 23 patients (46.0%) reported that they already had enough information, 12 (24.0%) that they did not have the ability to obtain additional information (e.g., no internet access), and 7 (14%) that they were not interested. Ten percent of patients stated that they did not want to get carried away by too much information. Seventy-seven patients (77.8%) reported that they knew about the Robert Koch Institute; however, only 66 patients (66.7%) could describe its function correctly.

### 3.3. Attitudes

Most patients (94.9%) stated that the virus was dangerous (in general and/or to them personally). Their most common reason (in approximately one third of patients) was that they saw themselves as patients at risk. Between 13.8% and 17.0% attributed danger to the deadly course of the disease, the lack of any specific cure, and the lack of knowledge about the virus, followed by other reasons, such as the high rate of infection.

Most patients (94.9%) felt that current restrictions on everyday life (social distancing, etc.) were necessary (94.9%). Most patients (94.9%) felt that these measures to contain the virus were sufficient, and 4.0% felt that they were excessive. Ninety-five percent stated that they understood why the measures were necessary, and 70% gave the correct reasons for the preventive measures (e.g., reducing the distribution of the virus or “flattening the curve”).

### 3.4. Practices

**Figure 2:**
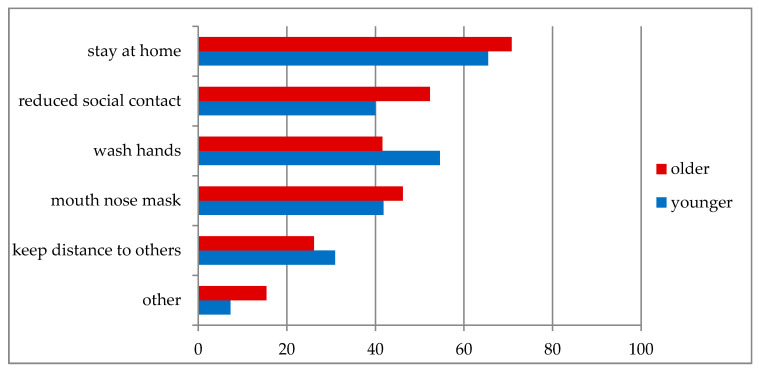
Actively reported preventive measures in response to COVID-19 (%).

Almost two thirds of the patients (61.6%) were recommended to stay at home more often by their family members. Seventy-two patients (72.7%) reported that they had changed their behavior since the appearance of the virus; only 4.0% reported taking no measures to protect themselves. The most common actively reported preventive behaviors are shown in Figure 2. Active reporting means that patients were not given a choice of answers but were free to choose their own.

Others include increased use of disinfectants (1), separation of towels (1), avoid public transport (1), and additional vaccination (1). Active reporting means that patients were not given a choice of answers but were free to choose their own.

When asked “Have you reduced social contacts?”, 86.9% of patients stated that they had reduced their contacts with relatives and friends and stayed at home more. Among patients who had not reduced their contacts, most stated that they had only a few contacts already and/or that they had to stay home anyway, mostly due to their Parkinson’s disease.

When asked about contacts with their relatives and friends, 77 patients (77.8%) reported that they had contact with their families at least several times a week, and 40 patients (40.4%) reported that they had a similar amount of contact with their friends. Thirty-nine patients (39.4%) saw their family members face-to-face, 42 (42.4%) only talked to them by telephone, and 16 (16.2%) used more up-to-date means of communication, such as video telephony and smartphone messenger services. The majority of patients (61.6%) kept in touch with friends by telephone.

Among the 73 patients with grandchildren (73.7%), 61 patients (83.6%) did not have contact with their grandchildren at present (including 8 who did not have contact with them before the coronavirus). The discontinuation of contact was mainly proposed by the parents of the grandchildren, the patients themselves, or both (86.8%). Thirty-six (73.6%) of those who did not have contact with their grandchildren stated that they suffered from this.

### 3.5. Burdens and Restrictions

Anxiety and worries about the present situation were reported by 58 patients (58.6%). Their greatest fear was infection with the coronavirus. In addition, the general uncertainty, the economic and social developments, and the possible loss of other medical care worried the patients.

Restrictions on everyday life due to the coronavirus were reported by 54 patients (54.5%). As physical burdens, lack of outdoor activities (10 patients [10.1%]), worsening of their Parkinson’s disease (19 patients [19.2%]), and further illness or injury were mentioned. As mental stresses, 22 patients (22.2%) mentioned the lack of social contacts and the ability to go outside, 10 (10.1%) were afraid of becoming infected, and 32 (32.3%) were burdened by other fears and worries.

On the physical level, 31 patients (31.3%) complained about a decrease in their mobility since the beginning of the restrictions, mainly because of worsening of their Parkinson’s disease and a lack of treatment. Consistent with this, many prescribed therapies had been canceled (63.2% of physiotherapy, 57.5% of occupational therapy, 62.5 of speech therapy). Thirty-four patients (34.3%) also reported that they had to refrain from sports activities (such as sports therapy, but also walking outside, or sports groups). However, 28 of these 34 patients (82.4%) found alternative forms of activity by doing their exercises at home or going outside.

### 3.6. Association between KAP and Clinical Parameters

Older patients with Parkinson’s disease had lower MoCa scores and higher MDS-UPDRS III scores than younger patients, indicating poorer cognitive function and greater motor impairment (Table 1). Older and younger patients did not differ in BDI, SAMS, or NMS-Q scores (Table 1).

The items for KAP and burden with respect to COVID-19 did not differ between younger and older patients (Table 2). Compared with control subjects, more patients with Parkinson’s disease (younger and older) reported having actively sought information (question 6). In comparison to the controls, more patients with Parkinson’s disease (younger, older and entire group with Parkinson’s disease) believed that the virus was dangerous (question 8). The other items for KAP and burden with respect to COVID-19 did not differ between patients with Parkinson’s disease and controls. Most items of KAP and burden did not differ between male and female patients; however, female patients more frequently perceived the current situation as threatening (*p* = 0.014, chi-square test).

### 3.7. Adherence to Preventive Measures

Knowledge and attitudes are crucial elements of adherent behavior. We observed some discrepancies between answers to simple yes/no questions and to detailed qualitative questions, indicating that around 30% of patients were nonadherent. Although 86 patients (89.6%) felt that they were sufficiently to very well informed about preventive measures and COVID-19, 25 of these patients (29.1%) were not able to correctly describe the function of the Robert Koch Institute. This is surprising, because the Robert Koch Institute was omnipresent in the media during the time of the study. It is Germany’s national public health institute in the field of surveillance, control, and prevention of diseases, comparable to the US Centers for Disease Control and Prevention. Although 77 patients (77.8%) reported that they knew what the Robert Koch Institute was, the qualitative question showed that only 67 (67.7%) were able to correctly name one of its functions. Thirty patients (29.7%) were not able to name a correct aim of the current restrictions, and this was also associated with limited knowledge about the Robert Koch Institute (*p* = 0.04, chi-square test). Fourteen patients (13.6%) did not know what the Robert Koch Institute was nor why the measures were taken. Missing knowledge about the Robert Koch Institute or the aim of the measures was not associated with educational level (*p* = 0.08, *p* = 0.133, respectively), MoCa score (*p* = 0.13, *p* = 0.38, respectively), BDI score (*p* = 0.81, *p* = 0.62, respectively), or MDS-UPDRS III score (*p* = 0.14, *p* = 0.57, respectively). Patients with limited knowledge were older (mean age, 74.4 years; SD = 7.2) than patients with good knowledge (mean age, 70.0 years; SD = 8.5) (*p* = 0.03). Patients who felt that they were very well informed about COVID-19 were more likely to search actively for further information (*p* = 0.03) than patients who felt that they were not sufficiently informed about COVID-19; in contrast, none of the five patients who felt that they were poorly informed about COVID-19 actively searched for information.

Ninety-five percent of patients believed that the virus was dangerous, mainly because they regarded themselves as being at higher risk. Ninety-five percent of patients also perceived the current restrictions on everyday life to be necessary or sufficient. Approximately 60% perceived the current situation as threatening. Perceiving the situation as threatening was not associated with regarding the virus as dangerous (*p* = 0.58), actively searching for further information (*p* = 0.20), general changes in behavior (*p* = 0.09), or reducing social contact (*p* = 0.30).

Seventy-two patients (72.7%) reported that they had changed their behavior (question 10), and 99% reported at least one specific preventive behavior such as washing hands or social distancing. Eighty-six patients (86.9%) reported that they had reduced social contacts and stayed at home. However, 27 (27.3%) of the patients (who did not live together with their families in one house) had personal contact with their relatives several times a week. Patients who did not reduce personal contact with relatives were significantly more likely to have only moderate or poor knowledge of COVID-19 (*p* = 0.003). Of note, six patients (6.1%) who had good knowledge were nonadherent and did not reduce their social contacts. The SAMS score was weakly correlated with the number of preventive practices performed by the patients (*r* = −0.19, *p* = 0.037). This means that adherent patients (lower SAMS) took more measures to protect themselves and others from the virus.

## 4. Discussion

In this study, the KAP of patients with and without Parkinson’s disease was analyzed by means of semi-structured telephone interviews to estimate the extent of nonadherence to preventive measures.

The majority of patients with Parkinson’s disease understood the containment measures in the context of the coronavirus pandemic and felt sufficiently informed. Most patients informed themselves via television news, which is in line with the study by Prasad et al. [9]. In addition, younger patients tended to inform themselves via the internet, and older patients got their information from the radio or from family members. A closer analysis shows, however, that only about 30% of patients could name the correct function of the Robert Koch Institute, although it had been present daily in the most diverse media since the outbreak of the coronavirus pandemic in Germany. Moreover, 30% of patients could not explain why specific measures (such as frequent handwashing) were necessary. We therefore conclude that about 30% of the patients had an insufficient level of knowledge, although all patients indicated that they obtained information from various media. A low level of knowledge was not associated with a lower educational level, cognitive disorders, or depression.

With regard to attitudes, the picture is more homogeneous. Almost all patients stated that they considered the virus to be dangerous and that the preventive measures were reasonable. However, this attitude did not seem to be transformed into corresponding adherent behavior. On the basis of the simple yes/no questions (e.g., “Have you reduced contact?”, “Have you changed your behavior?”), the majority of patients seemed to be adherent and to adhere to the instructions. This was also the case in the recent study by Prasad et al. [9] in which all patients reported following “any preventive measure” against COVID-19 and all patients reported performing social distancing. However, if one adds the qualitative questions, as in our study, the reported high level of adherence is put into perspective. Although almost all of our patients claimed to have reduced contact, a closer analysis of the qualitative answers shows that a considerable proportion of the patients continued to meet relatives in person almost daily. This shows that the use of a simple dichotomous question (“Have you reduced social contact?”) overestimates adherence to this preventive measure. Such simply structured queries are rather unsuitable for patients with Parkinson’s disease. On the other hand, it is alarming that 27% of patients did not adhere to the important preventive measure of contact reduction. In general, patients with good drug adherence (according to the SAMS) also seemed to practise more preventive measures (e.g., handwashing, mouth and nose protection) than did nonadherent patients. However, adherence to the measure of contact reduction was also related to knowledge. Patients who did not reduce personal contact with relatives had significantly less knowledge. On the other hand, there were also patients who did not reduce social contact against their better knowledge. This means that both intentional and unintentional nonadherence play a role.

In terms of burden, anxiety and worries about the current situation were reported by 58.6% of patients, consisting of fear of becoming infected as well as worries about future economic and social development. In the study by Prasad et al., only 11% of patients reported worsening of Parkinson’s disease symptoms following the onset of the COVID-19 pandemic [9]. In contrast, in our study, 31.3% of the patients complained about a decrease in their mobility since the beginning of the restrictions, mainly due to worsening of their Parkinson’s disease and cancelation of their regular therapies (e.g., physiotherapy). The discrepancy between these studies may be partly explained by differences in the degrees of preventive measures in Germany and India and the younger age of the patients in the study by Prasad et al. Within the framework of this telephone survey, we did not want to overburden the patients with too many questionnaires. However, it would be of great interest in further studies to investigate how anxiety, depression and psychotic symptoms appear in patients with Parkinson’s disease before and after COVID-19. For this purpose, disease-specific questionnaires would have to be used.

The crucial question is, what measures can improve KAP and adherence in elderly people? This study cannot provide a conclusive answer to this question. Some preliminary conclusions can nevertheless be drawn. First of all, it is crucial to know how (i.e., on which channels) we reach older people in the first place. The most important sources of information are news (television, radio, newspaper) and relatives. Direct communication via social media is therefore not very effective. Social media would only reach older people indirectly at most if younger relatives were informed via these channels. Despite the fact that almost everyone stated that they knew something about the pandemic (some even found the wealth of information too great), it became apparent that the purpose of the measures was not always understood through the existing communication channels. Perhaps it would make more sense at this point to communicate the measures in a form that allows interaction between the communication partners, e.g., via telephone consultation or personal contact (with appropriate preventive measures).

The present study has several limitations. The patients were recruited from a specialized neurological hospital, and the interviews were performed in a short time interval during the ongoing pandemic. This limits the generalizability of the results. On the other hand, it is important to keep in mind that simple dichotomous questions cannot provide a valid estimate of true adherence to preventive measures. Because of the restrictions, it was not possible to provide current clinical characteristics of the cohort by personal assessments. The clinical data provided, such as MDS-UPDRS III scores, were obtained between two and six months before the interviews were performed and can therefore only provide an estimate of the characteristics of the cohort. Nevertheless, we decided to provide these clinical data, because we assume that dramatic changes in MDS-UPDRS III scores did not occur during a few months for the whole cohort. Moreover, we think that this may help to make the cohort comparable to other Parkinson’s disease cohorts. It remains important to note that the results for some items (e.g., how do patients receive information?; what preventive measures do they perform?) are based on actively reported answers. We did not provide a selection of answers (multiple-choice) in order to avoid answers according to social desirability and in order to get an impression of what was truly prominent in the minds of the interviewed patients.

## Figures and Tables

**Table 1:** Clinical and demographic characteristics.

		Control		PD Older		PD Younger		
		n	%	n	%	n	%	*p*
Gender	female	6	28.6	22	38.6	13	31.0	0.612
	male	15	71.4	35	61.4	29	69.0	
Marital state	married	15	71.4	47	83.9	33	80.5	0.077
	divorced/widowed	2	9.5	7	12.	7	17.1	
	single	4	19.0	2	3.6	1	2.4	
Living situation	with others/relatives	15	71.4	48	84.2	37	88.1	0.239
	alone	6	28.6	9	15.8	5	11.9	
Education level	low	8 _a_	38.1	13 _a_	22.8	6 _a_	14.3	0.011
	middle	6 _a,b_	28.6	13 _a_	22.8	22 _b_	52.4	
	high	7 _a_	33.3	31 _a_	54.4	14 _a_	33.3	
Occupation	pensioned	13 _a_	61.9	57	100.0	38 _b_	90.5	<0.001
	employed	7 _a_	33.3	0	0.0	2 _b_	4.8	
	unemployed	1 _a_	4.8	0	0.0	2 _a_	4.8	
		M	95%CI	M	95%CI	M	95%CI	
Age (years) ^+^		68.0	64.0, 78.0	78.0	77.0, 79.0	65.5	63.0, 68.0	<0.001
BDI ^+^		7.0	3.0, 9.0	9.0	8.0, 13.0	11.0	10.0, 14.0	0.001
MoCa ^+^		24.0	23.0, 26.0	22.0	22.0, 23.0	25.0	25.0, 27.0	<0.001
MDS-UPDRS III ^+^				30.0	30.0, 38.0	22.0	21.0, 33.0	0.06
NMSQ *				10.4	9.0, 11.7	10.5	9.2, 11.9	0.87
SAMS ^+^		5.0	5.0, 8.0	6.0	4.0, 9.0	5.0	4.0, 9.0	0.69

+ Mean and 95%CI; * Median and 95%CI; Values in the same row and sub-table where the sub-script is not identical differ at *p* < 0.05 (Anova or Kruskal-Wallis for metric values and chi-square test for nominal values, with Bonferroni correction).

**Table 2:** Group comparisons KAP and burden.

		Control		PD Older		PD Younger		
		n	%	n	%	n	%	*p*
Knowledge								
Information	Very well-informed	10 _a_	47.6	19 _a_	35.2	13 _a_	31.0	0.022
	Well-informed	5 _a_	23.8	21 _a_	38.9	17 _a_	40.5	
	Sufficiently informed	0	0.0	11 _a_	20.4	5 _a_	11.9	
	Poorly informed	1 _a_	4.8	3 _a_	5.6	2 _a_	4.8	
	Excessively informed	5 _a_	23.8	0 _b_	0.0	5 _a_	11.9	
Active search for additional information	Yes	6 _a_	28.6	22 _a,b_	39.3	25 _b_	61.0	0.027
	No	15 _a_	71.4	34 _a,b_	60.9	16 _b_	39.0	
Advice from family to remain at home	Yes	14	77.8	37	67.3	24	57.1	0.427
	No	4	22.2	17	30.9	18	42.9	
	No Family	0	0.0	1	1.8	0	0.0	
Familiar with RKI	Yes	15 _a,b_	71.4	33 _a_	57.9	34 _b_	81.0	0.048
	No	6 _a,b_	28.6	24 _a_	42.1	8 _b_	19.0	
Can correctly describe aims of measures	Yes	10	47.6	39	68.4	30	71.4	0.146
	No	11	52.4	18	31.6	12	28.6	
Attitude								
Virus perceived as dangerous	Yes	14 _a_	66.7	55 _b_	96.5	39 _b_	97.5	0.000
	No	7 _a_	33.3	2 _b_	3.5	1 _b_	2.5	
Current restrictions perceived as	Necessary	16	76.2	47	85.5	34	81.0	0.670
	Sufficient	4	19.0	7	12.7	5	11.9	
	Excessive	1	4.8	1	1.8	3	7.1	
Situation perceived as threatening	Yes	12	57.1	37	67.3	26	65.0	0.710
	No	9	42.9	18	32.7	14	35.0	
Reporting of fears/worries	Yes	12	57.1	32	56.1	26	61.9	0.656
	No	9	42.9	23	40.4	16	38.1	
Practices								
Change in behaviour	Yes	18	85.7	38	67.9	34	81.0	0.162
	No	3	14.3	18	32.1	8	19.0	
Reduction of social contacts/remaining at home	Yes	21	100.0	48	87.3	38	90.5	0.233
	No	0	0.0	7	12.7	4	9.5	
Grandchildren	Yes	14	66.7	44	80.0	29	70.7	0.396
	No	7	33.3	11	20.0	12	29.3	
Personal contact with Grandchildren	Yes	1	7.7	6 _a_	13.6	6	20.7	0.390
	No	11	84.6	31	70.5	22	75.9	
	No regardless of corona virus	1	7.7	7	15.9	1	3.4	
Burden								
Affliction because of discontinued contact	Yes	9	64.3	22	50.0	17	58.6	0.807
	No	3	21.4	9	20.5	5	17.2	
Daily life restricted	Yes	14	66.7	27	49.1	27	64.3	0.212
	No	7	33.3	28	50.9	15	35.7	
Mobility deteriorated	Yes	3	14.3	15	27.3	16	38.1	0.136
	No	18	85.7	40	72.7	26	61.9	

Values in the same row and sub-table where the sub-script (a, b) is not identical differ at *p* < 0.05. In sub-tables with an expected cell frequency <5 the exact test (Monte Carlo) was used.

## Supplementary Materials

The following are available online at https://www.mdpi.com/2077-0383/9/6/1643/s1, Table S1: The questionnaire.
