# Supplementary material for: Knowledge, Attitudes, Practices, and Burden During the COVID-19 Pandemic in People with Parkinson’s Disease in Germany
Source: J Clin Med. 2020 May 29;9(6):1643. doi: 10.3390/jcm9061643 (PMC7355903; doi:10.3390/jcm9061643)
Supplement: Supplementary file 1 [file jcm-09-01643-s001.pdf]

Supplement

Table 1. Interview

1. Have you been tested positive for Corona?
   (1) Yes (2) No (3) test results pending
2. Have you been in contact with a person who was tested positive for Corona?
   (1) Yes (2) No
   If (1) yes: relationship to infected person
3. Are you currently/have you been in quarantine?
   (1) Yes (2) No
   If (1) Yes: when?
4. How well-informed do you personally feel about Corona?
   (1) Very well (2) well (3) sufficiently (4) poorly (5) excessively
5. From where do you obtain your information about Corona?
6. Do you actively search for additional information regarding Corona?
   (1) Yes (2) No
   If (2) No: why not?
7. Are you familiar with the Robert Koch Institute (RKI) and its function?
   (1) Yes (2) No
   If yes, additional information on the attributed function of the RKI was noted
8. Do you believe that the virus is dangerous (for you personally and on a general level)?
   (1) Yes (2) No
   Why?
9. Do you perceive the current restrictions in everyday life as
   (1) necessary (2) sufficient (3) excessive?
   Can you understand why those restrictions were imposed?
   (1) Yes (2) No
   Why?
10. Have you changed your own behavior since the appearance of the virus?
    (1) Yes (2) No
11. Which measures do you personally take to protect yourself from an infection with Corona?
12. Do you remain at home as far as possible and have you reduced your social contacts?
    (1) Yes (2) No
    If (2) No: Why?
13. When was the last time you were in contact with (a) family (b) friends/acquaintances? In what form did the contact take place? (e.g. personal contact, contact via telephone/skype/letters)
14. Did your family members advise you to remain at home in the current situation?
    (1) Yes (2) No (3) No Family
15. Do you have grandchildren?
    (1) Yes (2) No
    If (1) Yes, are you still in personal contact with them? (1) Yes (2) No (3) No contact regardless of Corona
    If (2) No: Who suggested discontinuing the contact? (1) the grandchild's parents (2) the grandchild (3) the patient (4) collective decision (5) other
    Does the discontinued contact afflict you? (1) Yes (2) No
16. Do you perceive the current situation as threatening?
    (1) Yes (2) No
    Is there anything in particular that frightens you? (1) Yes (2) No
    If (1) Yes: What?
17. Do you feel restricted in your daily life because of Corona?

(1) Yes (2) No

18. What afflicts/restricts you most...
    (a) physically
    (b) psychologically
19. Has your mobility deteriorated in the last weeks because of the current regulations?
    (1) Yes (2) No
    If (1) Yes: how so?
20. Do your still have therapy [(a) Physiotherapy (b) occupational therapy (c) speech therapy]?
    (1) Yes (2) No, cancelled due to regulations (3) No, cancelled due to lack of motivation (4) no therapy prescribed
    If (1) Yes, how often?
21. Which of your usual physical activities are you unable to perform due to the current regulations?
22. Which alternatives have you found to substitute the physical activities you can no longer perform because of the current regulations?
